# Mating changes the subcellular distribution and the functionality of estrogen receptors in the rat oviduct

**DOI:** 10.1186/1477-7827-7-139

**Published:** 2009-11-30

**Authors:** Pedro A Orihuela, Lidia M Zuñiga, Mariana Rios, Alexis Parada-Bustamante, Walter D Sierralta, Luis A Velásquez, Horacio B Croxatto

**Affiliations:** 1 Laboratorio de Inmunología de la Reproducción, Facultad de Química y Biología, Universidad de Santiago de Chile, Santiago, Chile; 2 Centro para el Desarrollo en Nanociencia y Nanotecnología-CEDENNA, Santiago, Chile; 3 Millennium Institute for Fundamental and Applied Biology, Santiago, Chile; 4 Unidad de Reproducción y Desarrollo, Facultad de Ciencias Biológicas, Pontificia Universidad Católica de Chile, Santiago, Chile; 5 Instituto de Nutrición y Tecnología de los Alimentos, Universidad de Chile, Santiago, Chile

## Abstract

**Background:**

Mating changes the mode of action of 17beta-estradiol (E2) to accelerate oviductal egg transport from a nongenomic to a genomic mode, although in both pathways estrogen receptors (ER) are required. This change was designated as intracellular path shifting (IPS).

**Methods:**

Herein, we examined the subcellular distribution of ESR1 and ESR2 (formerly known as ER-alpha and ER-beta) in oviductal epithelial cells of rats on day 1 of cycle (C1) or pregnancy (P1) using immunoelectron microscopy for ESR1 and ESR2. The effect of mating on intraoviductal ESR1 or ESR2 signaling was then explored comparing the expression of E2-target genes c-fos, brain creatine kinase (Ckb) and calbindin 9 kDa (s100g) in rats on C1 or P1 treated with selective agonists for ESR1 (PPT) or ESR2 (DPN). The effect of ER agonists on egg transport was also evaluated on C1 or P1 rats.

**Results:**

Receptor immunoreactivity was associated with the nucleus, cytoplasm and plasma membrane of the epithelial cells. Mating affected the subcellular distribution of both receptors as well as the response to E2. In C1 and P1 rats, PPT increased Ckb while both agonists increased c-fos. DPN increased Ckb and s100g only in C1 and P1 rats, respectively. PPT accelerated egg transport in both groups and DPN accelerated egg transport only in C1 rats.

**Conclusion:**

Estrogen receptors present a subcellular distribution compatible with E2 genomic and nongenomic signaling in the oviductal epithelial cells of C1 and P1 although IPS occurs independently of changes in the distribution of ESR1 and ESR2 in the oviductal epithelial cells. Mating affected intraoviductal ER-signaling and induced loss of functional involvement of ESR2 on E2-induced accelerated egg transport. These findings reveal a profound influence on the ER signaling pathways exerted by mating in the oviduct.

## Background

It is well recognized that in the female mammal ovarian steroids, embryonic signals or mating-associated signals regulate egg transport through the oviduct (for review see [1]). In cyclic rats, oocytes reach the uterus approximately 72 h after ovulation, whereas in pregnant rats embryos take 96 h. Oocytes take 96 h to traverse the oviduct in rats made pseudopregnant by mechanical stimulation of the cervix in the evening of proestrus, therefore the above difference is dependent on mating-associated signals rather on whether eggs are fertilized or not [2]. Eggs cross the ampullary-isthmic junction 9 h earlier and egg surrogates move at higher speed in the isthmic segment with most of them arriving earlier to the intramural segment in pregnant rats than in cycling rats [3]. Thus, a broad change occurs in oviductal functioning elicited by mating-associated signals.

A single injection of 17β-estradiol (E_2_) on day 1 of the cycle or pregnancy shortens oviductal transport of eggs from the normal 72-96 h to less 24 h [1]. Previously, we demonstrated that inhibitors of RNA and protein synthesis block E_2_-induced oviductal embryo transport acceleration in pregnant rats, but fail to do so in cyclic rats [4,5]. Furthermore, in cyclic rats exogenous E_2 _activates protein phosphorylation in the oviduct via a nongenomic pathway, since such activation occurs when mRNA synthesis is completely suppressed by α-Amanitin [6]. Estradiol-induced phosphorylation is essential for its effect on oocyte transport in cycling rats since local administration of a broad-spectrum inhibitor of protein kinases totally blocks the effect of E_2 _on egg transport [7,8]. Thus, E_2 _accelerates oviductal egg transport through nongenomic pathways in cyclic rats, while it does it through genomic pathways in pregnant rats. Recently, this change in pathways has been designated "intracellular path shifting" (IPS) [9]. Further investigation has shown that activity of the enzyme Catechol-O-Methyltransferase (COMT) is higher in the oviduct of cyclic than pregnant rats while OR486 a selective inhibitor of COMT blocked the effect of E_2 _on oviductal egg transport only in cyclic rats suggesting that decreased activity of oviductal COMT induced by mating is one of the underlying mechanisms of IPS [9]. Although the physiological relevance of IPS has not been clearly established it is probable that decrease in the COMT activity induced by mating in the oviduct protects the embryos from the deleterious effect that methoxyestradiols exert during the first stages of development [10].

Estrogens induce cellular changes in their target organs through several different mechanisms that involve activation of estrogen receptors (ER). The two main forms of ER, ESR1 and ESR2 (formerly known as ER-α and ER-β), have distinct tissue expression patterns in both humans and rodents [11]. The antiestrogen ICI 182780 blocks E_2_-induced egg transport acceleration in cyclic and pregnant rats [7] indicating that ER participates in both the genomic and the nongenomic pathways involved in the kinetic action of E_2 _on the oviduct. However, we have found that levels of ESR1 and ESR2 mRNA and protein in oviducts of pregnant rats were similar to those oviducts of cycling rats, suggesting that IPS is not explained by changes in the expression of ER in the oviduct [12]. Herein, we determined the effect of mating on subcellular distribution and functionality of ESR1 and ESR2 in the rat oviduct. First, we compared immunoreactivity of both ER associated to cell membrane, cytoplasm and nucleus between epithelial cells of the ampullary and isthmic segments of cyclic and pregnant rats following treatment with E2. We also determined the effect of selective agonists for ESR1 (PPT) or ESR2 (DPN) on mRNA levels of three E2-inducible genes c-fos, brain creatine kinase (Ckb) and calbindin 9 kDa (s100g) [13,14] in the oviduct of pregnant and cyclic rats. Additionally, the role of ESR1 and ESR2 on oviductal egg transport was evaluated in cyclic or pregnant rats treated with PPT or DPN.

## Methods

### Animals

Sprague-Dawley rats (bred in house) weighing 200-260 g were used. Animals were kept under controlled temperature (21-24°C), and lights were on from 0700 to 2100 h. Water and pelleted food were supplied *ad libitum*. The phases of the estrous cycle were determined by daily vaginal smears [15] and all females were used after showing two consecutive 4-day cycles. Females in pro-estrus were kept either isolated or caged with fertile males. The following day (estrus) was designated as C1 in the first instance and day P1 in the second, provided spermatozoa were found in the vaginal smear. The care and manipulation of the animals was done in accordance with the ethical guidelines of our institution.

### Treatments

#### Systemic administration of E_2_

On C1 or P1 E_2 _5 μg was injected subcutaneously (s.c.) as a single dose dissolved in 0.1 mL propylene glycol. Control rats received propylene glycol alone.

#### Local administration of selective agonist of ESR1 (PPT) or ESR2 (DPN)

PPT (1,3,5-tris(4-hydroxyphenyl)-4-propyl-1H-pyrazole, Sigma Chem. CO, St. Louis, MO) [16] or DPN (Diarylpropionitrile, Tocris Cookson Inc. Ellisville, MO) [17] were injected into each bursa at a concentration of 7.5, 22.5 or 67.5 ng/μL in DMSO 1%. Control rats received the corresponding vehicle alone. Since the range of effective doses of PPT and DPN given systemically is between 2.5 μg/μL-250 μg/μL [18,19] we considered appropriate diminished these doses to approximately 1000-fold for local (intrabursal) injection. To our knowledge these doses of PPT or DPN did not change the plasmatic E_2 _and Progesterone level in the rat.

### Animal surgery and assessment of egg transport

Intrabursal administration of agonists, which minimizes the dose needed to affect the oviduct without systemic effects, was performed on C1 or P1 as previously described [5]. At this time, ovulation has already taken place, so this treatment cannot affect the number of oocytes ovulated. Egg transport was evaluated as previously published [2,4,5]. Twenty-four hours after treatment, animals were sacrificed and their oviducts were flushed individually with saline. Flushing was examined under low-power magnification (25×), and the number of eggs found was recorded.

### Real-time PCR

Whole oviducts on C1 (N = 4) or P1 (N = 4) were dissected and flushed to avoid contamination with oocytes or embryos mRNA. Oviductal RNA was isolated using Trizol Reagent (Invitrogen, Gaithersburg, MD) and 1 μg of total RNA of each sample was treated with Dnase I Amplification grade (Invitrogen). The single-strand cDNA was synthesized by reverse transcription using the Superscript III Reverse Transcriptase First Strand System for RT-PCR (Invitrogen), according to the manufacturer's protocol. The Light Cycler instrument (Roche Diagnostics, GmbH Mannheim, Germany) was used to quantify the relative gene expression of c-fos, Ckb or s100g in the oviduct of cyclic and pregnant rats; *Gapdh *was chosen as the housekeeping gene for load control because we have previously demonstrated that E_2 _or pregnancy did not affect its expression [20]. The SYBR^® ^Green I double-strand DNA binding dye (Roche Diagnostics) was the reagent of choice for these assays. Primers for *c-fos *were 5' CCG AGA TTG CCA ATC TAC TG 3' (sense) and 5' AGA AGG AAC CAG ACA GGT CC 3' (antisense), *Ckb *5' AAG CTG GCA GTA GAA GCC CT 3' (sense) 5' TTG TCG AAG AGG AAG TGG TC 3' (antisense), *s100g *5' GGC AGC ACT CAC TGA CAG C 3' (sense) 5' CAG TAG GTG GTG TCG GAG C 3'(antisense) and for *Gapdh *were 5' ACC ACA GTC CAT GCC ATC AC 3' (sense) and 5' TCC ACC ACC CTG TTG CTG TA 3' (antisense). The thermal cycling conditions included an initial activation step at 95°C for 25 min, followed by 40 cycles of denaturizing and annealing-amplification (95°C for 15 sec, 60°C for 15 sec and 72°C for 30 sec) and finally one cycle of melting (95° to 60°C). To verify specificity of the product, amplified products were subject to melting curve analysis as well as electrophoresis, and product sequencing was performed to confirm identity as described by Muscillo et al [21]. The expression of transcripts was determined using a method previously reported [22,9].

### Immunoelectron microscopy

Oviducts from vehicle and E_2_-treated rats were separated into ampulla and isthmus and the excess mucus was removed in each segment by flushing with 50 μL saline. Both segments were fixed in 4% freshly depolymerised paraformaldehyde, 0.5% glutaraldehyde in phosphate buffer pH 7.4 0.1 M containing saccharose 0.1 M, DMSO 1% and CaCl_2 _1% for 2-4 h at room temperature. The fixed samples were washed three times with phosphate buffer, dehydrated in a graded ethanol series and infiltrated with LR Gold (Plano, München, FRG). Subsequently, the samples were transferred to gelatine capsules filled with 0.8% (w/v) benzoyl peroxide in LR Gold and kept for polymerization at a pressure of 500 mmHg. The blocks were cured for 1-2 days at room temperature before sectioning with a Sorvall-2000 ultramicrotome using a diamond knife. The sections (50-80 nm) were mounted on formvar-coated nickel grids and incubated on droplets of 0.1 M glycine in PBS pH 7.6, and subsequently blocked with 1% bovine foetal serum for 2 h at room temperature. The grids were then incubated for 2 h with a rabbit anti-ESR1 (MC-20, Santa Cruz Biotechnology, Santa Cruz, CA) or anti-ESR2 (clone 68-4, Chemicon International, Billerica, MA) at 1:50 dilution. After washing with PBS, the preparations were incubated for 1 h with goat anti-rabbit immunoglobulin conjugated to 10 nm gold particles (Kirkegaard & Perry Laboratories Inc, Gaithersburg, MD) diluted 1:30. Sections were washed and contrasted with Reynolds stain [23]. All sections were examined using a Phillips-TECNAI 12 BIOTWIN EM Microscope (FEI Company, Hillsboro, OR) at 80 kV. As negative controls the primary antibody was replaced by rabbit preimmune serum or oviductal samples without prior incubation with anti-ESR1 antibody or anti-ESR2 antibody were also included. For further validation we also performed immunoelectron microscopy of the isthmic segment from vehicle and E_2_-treated rats using a mouse anti-ESR1 from another company (H-151, Calbiochem, La Jolla, CA), as primary antibody diluted 1:30. Furthermore, we used gold-labeled particles of 40 nm (Kirkegaard & Perry Laboratories Inc) to obtain photomicrographs at low magnification for show unspecific background labeling in the oviductal lumen. At least ten areas of 63 μm^2 ^from different epithelial cells and different sections of an oviduct were photographed and the photomicrographs were digitalized in an iBook computer (Apple Computer Inc, Cupertino, CA), and gold particles present only in the cells were counted using the image analysis software Adobe Photoshop 7.0 (Adobe Systems Inc, San Jose, CA) by an observer blinded to the treatment groups. The results of the immunolabeling are presented as the quotient of the number of gold particles present divided by the area and cell number inspected [24].

### Statistical analysis

The results are presented as mean ± SE. Overall analysis was done by Kruskal-Wallis test, followed by Mann-Whitney test for pair-wise comparisons when overall significance was detected.

## Results

### Distribution of ESR1 and ESR2 in epithelial cells of mated and non-mated rat oviducts treated with E_2_

**Figure 1:**
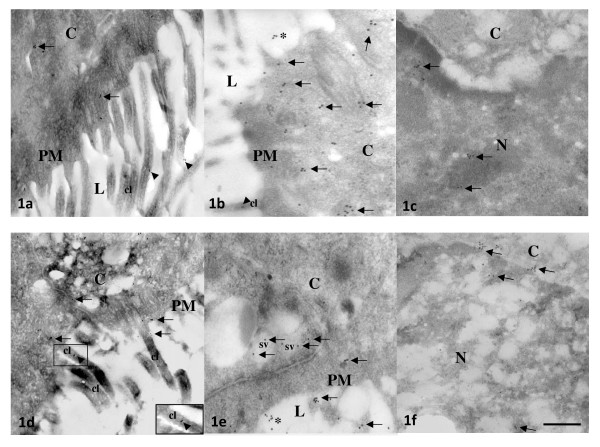
**Representative photomicrographs of oviductal epithelial cells processed by immunoelectron microscopy with gold labeled-antibodies of 10 nm for ESR1 (a-c) or ESR2 (d-f)**. Ampullary and isthmic sections of cyclic (**a-c**) and pregnant (**d-f**) rats are shown in the upper and lower panels, respectively. Arrows denotes ESR1-or ESR2 reacting gold particles in the epithelial cells. Arowheads emphasize the association of estradiol receptor immunoreactivity with cilia (**cl**, see insert in **d**). Asterisks indicate unspecific background laleling. Bar: 0.5 μm. **PM **= plasma membrane, **C **= cytoplasm, **N **= nucleus, **L **= lumen, **SV **= secretory vesicle.

**Figure 2:**
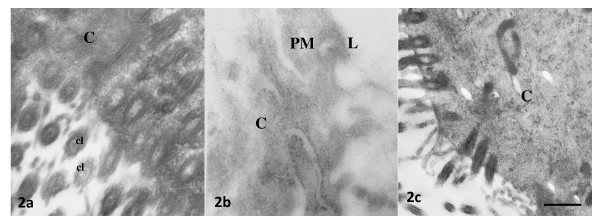
**Representative photomicrographs of oviductal epithelial cells processed by immunoelectron microscopy without prior incubation with anti-ESR1 antibody (a) or anti-ESR2 antibody (b), or incubated with rabbit preimmune serum (c)**. Bar: 0.5 μm. **PM **= plasma membrane, **C **= cytoplasm, **L **= lumen, **cl **= cilia. Bar: 0.5 μm.

**Figure 3:**
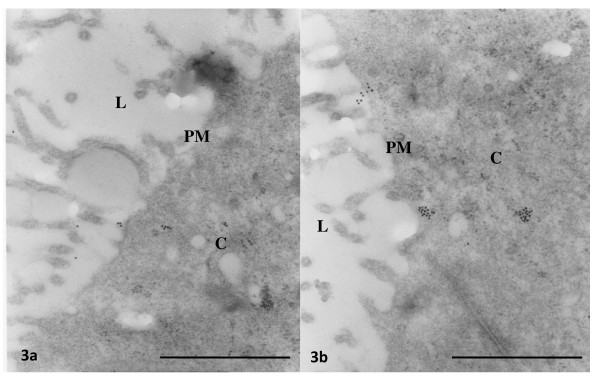
**Representative photomicrographs at low magnification of oviductal epithelial cells processed by immunoelectron microscopy with gold labelled-antibodies of 40 nm for ESR1 (a) or ESR2 (b)**. Arrows denotes ESR1-or ESR2 reacting gold particles in the epithelial cells. Note scarce unspecific background laleling. Bar: 0.5 μm. **PM **= plasma membrane, **C **= cytoplasm, **L **= lumen.

**Figure 4:**
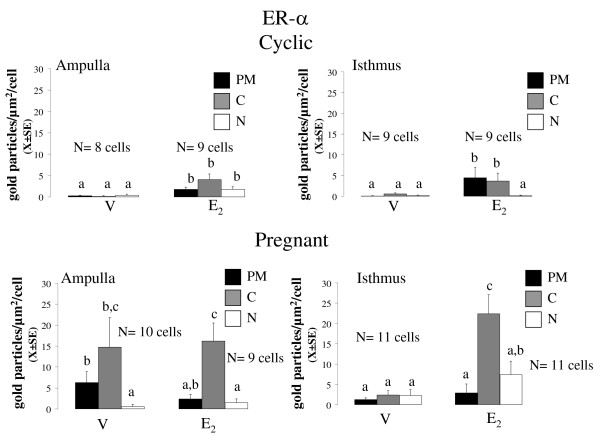
**Mean number of the density of gold particles (X ± SE) observed for ESR1 in the plasma membrane (PM), cytoplasm (C) and nucleus (N) of epithelial cells of oviductal ampulla (A) and isthmus (I) from rats on day 1 of the cycle or pregnancy, 3 hours after treatment with oestradiol**. Means with different letters are significantly different from each other within each graph (P < 0.05). a ≠ b ≠ c. Replicas of this experiment are stated in the figure.

**Figure 5:**
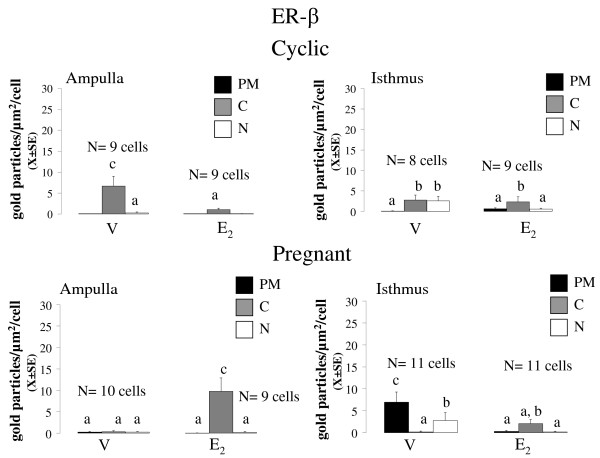
**Mean number of the density of gold particles (X ± SE) observed for ESR2 in the plasma membrane (PM), cytoplasm (C) and nucleus (N) of epithelial cells of oviductal ampulla (A) and isthmus (I) from rats on day 1 of the cycle or pregnancy, 3 hours after treatment with oestradiol**. Means with different letters are significantly different from each other within each graph (P < 0.05). a ≠ b ≠ c. Replicas of this experiment are stated in the figure.

At 09:00 h of C1 or P1, 8 rats were injected with E_2 _5 μg or vehicle and 3 h later they were sacrificed and their oviducts were separated into ampulla and isthmus and processed for immunoelectron microscopy. Representative photomicrographs of the subcellular distribution of ESR1 and ESR2-reacting gold particles in oviductal epithelial cells of the rat are shown in Figures 1, 2 and 3. Receptor immunoreactivity was found associated with the nucleus, cytoplasm and plasma membrane, including cilia, of the epithelial cells. Data for subcellular distribution of ESR1 in ampulla and isthmus are shown in Figure 4. Mating increased the immunoreactivity of ESR1 in the plasma membrane and cytoplasm of the ampullary segment although it did not affect the ESR1 immunoreactivity in the isthmus. In cyclic rats, E_2 _treatment increased the density of ESR1 labeling in all three compartments from both segments, except the nucleus of the isthmic segment, whereas in pregnant rats a major increase in labeling was observed only in the cytoplasm of the isthmic segment. Mating decreased immunoreactivity of ESR2 in the cytoplasm of the ampullary and isthmic segments although it increased labeling of ESR2 in the plasma membrane of the isthmus. In cyclic rats, E_2 _decreased ESR2 density in the cytoplasm in the ampulla and in the nucleus of the isthmus, whereas in pregnant rats, E_2 _increased the density of ESR2 labeling in the cytoplasm in the ampulla and decreased it in the plasma membrane and nucleus in the isthmus (Figure 5). The results using the mouse anti-ESR1 were similar to those obtained with the rabbit anti-ESR1 (not shown). Furthermore, low unspecific background labeling of gold particles was found in the lumen of epithelial cells in control experiments without primary antibody or incubation with rabbit preimmune serum (see figures 2 and 3). All this supports the specificity in the recognition of ER immunoreactivity.

### Effect of selective agonist of ESR1 or ESR2 on the level of *c-fos*, *Ckb *and *s100g *in the oviduct of pregnant and cycle rats

**Figure 6:**
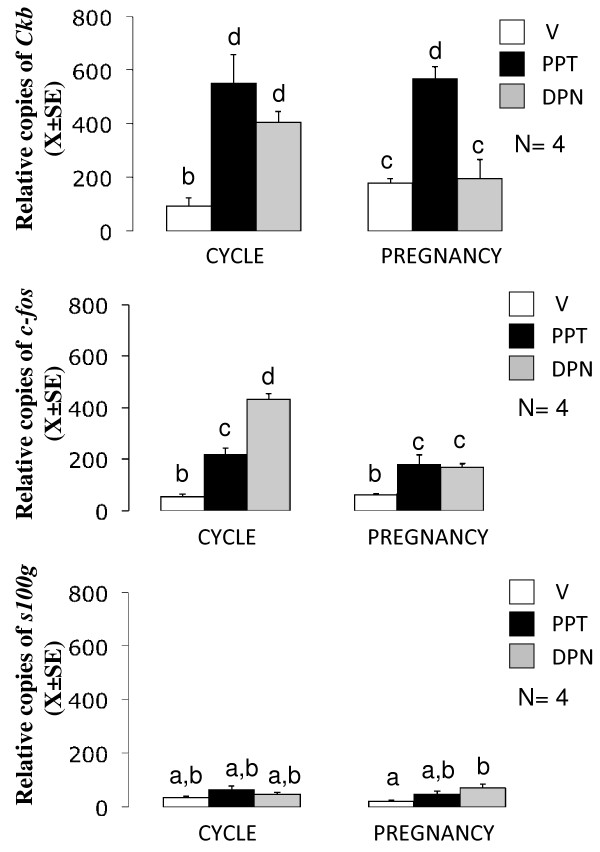
**Mean number of the relative copies (X ± SE) of *Ckb*, *c-fos *and *s100g *in the rat oviducts on day 1 of cycle or pregnancy following intrabursal treatments with the selective agonists of ESR1, PPT or ESR2, DPN**. V: vehicle of drugs, PPT: 67.5 ng/μL, DPN: 67.5 ng/μL. All treatments were given 3 h before autopsy. Each experiment consisted of 4 replicas. Means with different letters are significantly different from each other (P < 0.05), a ≠ b ≠ c ≠ d.

Rats on C1 (N = 4) or P1 (N = 4) were locally treated with 67.5 ng/μL of PPT or DPN and 3 h later oviducts were excised and their total RNA were processed by RT-PCR using specific primers for c-fos, Ckb, s100g or *Gapdh *as described above. Figure 6 shows that in cyclic rats oviductal levels of *c-fos*, *Ckb *and *s100g *were similar while in pregnant rats levels of *Ckb *were major than *c-fos *and *s100g*. PPT increased 5-fold and 3-fold the levels of Ckb and c-fos in cyclic and pregnant rats while s100g was not affected in both conditions. In cyclic rats, DPN increased 4- and 5-fold Ckb and c-fos respectively, while in pregnant rats DPN increased 2.5 fold c-fos and 2-fold s100g.

### Effect of selective agonist of ESR1 or ESR2 on oviductal egg transport in mated and non-mated rats

**Figure 7:**
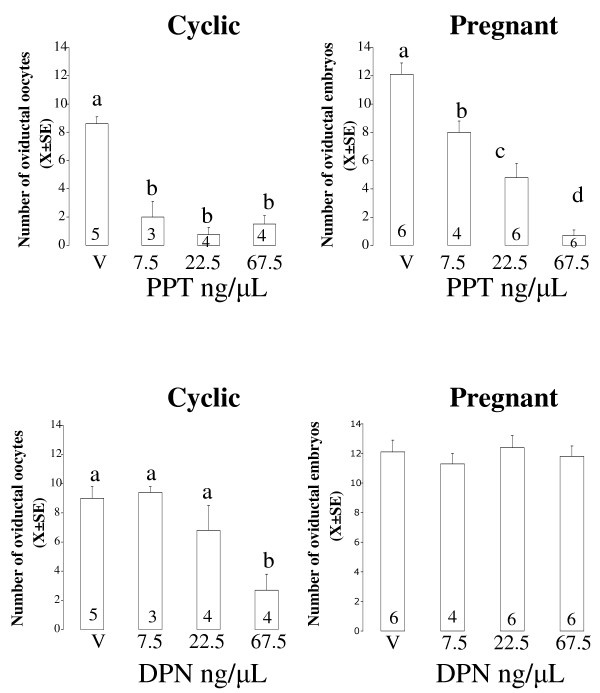
**Mean number of eggs (X ± SE) recovered from rat oviducts on day 2 of the cycle or pregnancy, 24 h after intrabursal treatment with different doses of the selective agonists of ESR1, PPT or ESR2, DPN**. Figures inside the bars indicate the number of animals used. Means with different letters are significantly different from each other within each graph, a ≠ b ≠ c (P < 0.05).

Rats on C1 or P1 were locally treated with PPT or DPN 7.5, 22.5 or 67.5 ng/μL and 24 h after treatment egg transport was evaluated in all groups as described above. The mean number (X ± SE) of eggs recovered from the oviducts of control or treated groups are shown in figure 7. Intrabursal administration of PPT decreased the number of eggs recovered from the oviduct in cyclic and pregnant rats although at lower doses in cyclic rats. Administration of DPN decreased the number of eggs only in C1, but not in P1 rats.

## Discussion

An important variable that influences E_2 _actions on its target cells is the differential distribution of ESR1 and ESR2. Previous works have reported presence of ESR1 and ESR2 in the epithelium and smooth muscle layers of the ampullary and isthmic segments of the rat oviduct [25,26]. Here, we show for the first time that ESR1 and ESR2 were found associated to the plasma membrane, cytoplasm and nucleus of the epithelial cells of both oviductal segments in the rat. This is in agreement with previous immunoelectron microscopy studies reporting localization of ESR1 in non-nuclear sites in other cell types [27,28]. Other studies have also shown the presence of ESR1 and ESR2 in non-nuclear sites of various cell types using western blot or ligand-blot of subcellular enriched fractions [29-31]. Ligand activation of ER associated to cell membrane and cytoplasm can modulate downstream pathways that induce discrete signaling responses, including stimulation of adenylyl cyclase in breast and vascular tissues [32,33], activation of Ca^2+ ^flux in arterial smooth muscle [34] or the cascade Src/Ras/ERK [35]. Our findings provide evidence of the presence of multiple pools of ER that could initiate genomic and nongenomic responses to E_2_. Furthermore, the data reported here show a dynamic behavior of these ER pools in response to mating-associated signals and to an E_2 _pulse.

Since mating induces IPS we expected that pregnant and cyclic rats would exhibit different ER subcellular distribution, e.g. higher ESR1, ESR2 or both in the nuclear compartment. However, quantitative analyses showed that mating increased the number of both receptors only in the non-nuclear compartments. Furthermore, when E_2 _was administered to pregnant or cyclic rats receptor immunoreactivity also accumulated in the non-nuclear compartments. Thus, IPS occurs independently of the changes in the distribution of ESR1 and ESR2 in the oviductal epithelial cells induced by mating. ESR1 and ESR2 are also expressed in the mucosa and muscle layer of the rat oviduct [25,26] so that it is possible that mating stimulates accumulation of ER in the nuclear compartment of other cell types. The changes described in gold particle density may reflect either change in antibody accessibility to immunoreactive epitopes or true changes in the expression level of ER. The current data does not allow to distinguish between these two possibilities but increases up to ten fold 3 hours after E_2 _administration seem more plausible as a result of ER dissociation from scaffolding proteins than a result of de novo synthesis. In fact, previously we have found that levels of ESR1 and ESR2 mRNA and protein in whole oviducts of pregnant rats were similar to those in oviducts of cycling rats, suggesting that mating does not regulate global expression of ER in the oviduct [12].

We observed different responses in the subcellular distribution of ER in the epithelial cells of the two oviductal segments. It is known that the relative proportion between ciliated and secretory epithelial cells varies considerably from ampulla to isthmus [36] so that is probable that mating-associated signals may have acted differentially on these two cell types. Further analysis that segregates the responses of ciliated from secretory epithelial needs to be done. It has been reported that in the rat, E_2 _acts only in the isthmic segment to accelerate egg transport [37] while that isthmus-specific apoptosis of epithelial cells and activation of cilia-localized ESR2A induced by clomiphene citrate act in parallel to block egg transport [19]. Thus, it is probable that differences in the distribution of ESR1 and ESR2 between ampulla and isthmus could reflect specific contribution of these segments to signals provided by E_2 _to regulate egg transport. Furthermore, we did not discard the possibility that E_2 _acts directly on the smooth muscle cells because it has been found presence of ESR1 and ESR2 in the myosalpinx of the rat [25,26].

Interestingly, ESR1 and ESR2 were also observed associated with the cilia of epithelial cells (see insert in figure 1d). Estradiol regulates differentiation and dedifferentiation of ciliated cells of the mammalian oviduct [38]. Furthermore, follicular fluid of human pre-ovulatory follicles containing high concentrations of estradiol and progesterone increased the ciliary beat frequency of human oviductal ciliated cells [39]. Our findings suggest that E_2 _could regulate ciliary activity directly through a nongenomic mechanism probably involving phosphorylation/dephosphorylation of some proteins (e.g. tubulin or dynein) present in this structure. Recently, it has been shown that ESR2 is colocalized with β-tubulin at stem portion of the cilia of the oviductal epithelial cells in immature rats [40]. Additionally, gold particles for ESR1 and ESR2 were found associated to secretory vesicles. This corroborates previous works reporting localization of ESR1 and ER2 in the rough endoplasmic reticulum and secretory vesicles of the female rat pituitary cells [41]. Although, the biological significance of the localization of ESR1 and ESR2 in secretory vesicles remains to be determined it is probable that a Golgi-dependent pathway could exist for translation of ER that could be translocated into the plasma membrane and mediate nongenomic responses [41].

In other estrogen-sensitive tissues ER subtype expression is differentially regulated by E_2_. In the human vena cava, E_2 _down-regulates ESR1 expression [42] while deprivation of E_2 _in the cerebral microvessels of ovariectomized rats is associated with a decrease in the expression of both isoforms and E_2 _replacement up-regulates ESR1 but does not affect expression of ESR2 [43]. In ovine endothelial cells, short-term treatment with E_2 _down-regulates ESR1, but not ESR2 while long-term treatment up-regulates ESR1 and down-regulates ESR2 expression [44]. Our findings provide the first evidence that E_2 _is able to differentially regulate not just the expression level, but also the subcellular distribution of ESR1 and ESR2 in a target cell. We also observed different responses in the expression of three E_2_-associated signaling genes, *c-fos*, *Ckb *and *s100g*, in the oviduct of pregnant and cyclic rats when ESR1 or ESR2 was activated. Moreover, activation of ESR1 or ESR2 increased expression of *c-fos *although mating only blunted the effect of ESR2. This indicates that mating-associated signals modulate intraoviductal signalling of both ER providing evidence that mating may change the functional role of these receptors in the rat oviduct. On the other hand, the role of *c-fos*, *Ckb *or *s100g *on IPS induction or E_2_-induced egg transport acceleration needs to be disclosed.

Given that PPT is 400-fold more selective and DPN is only 70-fold more selective for ESR1 an ESR2 respectively, it was not surprisingly that PPT would be more effective to accelerate oviductal egg transport than DPN in cyclic rats. However, mating decreased effectiveness of PPT and blocked the effect of DPN. Probably, the nongenomic pathway by which E_2 _accelerates egg transport operates through activation of either ESR1 and ESR2 while the genomic pathway only operates through ESR1. The fact that IPS is associated with suppression of ESR2 involvement in the kinetic effect of E_2 _in the oviduct indicate that mating exerts a profound influence on the biology of ER in a target organ of E_2 _that merits further investigation.

Shao et al [19] have reported that subcutaneous administration of DPN retard egg transport in the rat. In this study, immature animals were treated, prior to DPN administration, with gonatrophins to mimic the endogenous luteneizing hormone surge. Probably, this treatment could have affected the response of the oviduct to DPN. Another factor is that we recorded the number and distribution of eggs in the genital tract within the first 24 h after treatment. In order to detect whether PPT or DPN delay egg transport autopsies should be performed on day 4 or 5 of cycle or pregnancy respectively, but this was not done.

## Conclusion

Estrogen receptors ESR1 and ESR2 present a subcellular distribution in oviductal epithelial cells that is compatible with genomic and nongenomic actions of E_2 _in the rat oviduct. Mating is associated with changes in the basal and E_2_-induced subcellular distribution of ESR1 and ESR2 in these cells although it did not clearly explain IPS. Furthermore, mating affected signaling of both ER in the oviduct and induced loss of functional involvement of ESR2 on E_2_-induced accelerated egg transport. These findings reveal a profound influence on the intraoviductal ER signalling pathways exerted by mating.

## Competing interests

The authors declare that there is no conflict of interest that could be perceived as prejudicing the impartiality of the research reported.

## Authors' contributions

PAO participated in the design of the study, in directing and completing all experimental analysis and in writing the manuscript. LMZ, MR, APB performed the sampling of the animals, carried out the Real-Time PCR, intrabursal injections of drugs and assessment of the egg transport. WDS collaborate in the design of the studies of the immunomicroscopy of ESR1 and ESR2 and quantification of gold particles for ESR1 and ESR2. LV and HBC participated in planning experiments and contributed to drafting the manuscript. All authors have read and approved the final manuscript.
